# Supplementary material for: Lifestyle, sick leave and work ability among Norwegian employees with asthma—A population-based cross-sectional survey conducted in Telemark County, Norway
Source: PLoS One. 2020 Apr 17;15(4):e0231710. doi: 10.1371/journal.pone.0231710 (PMC7164599; doi:10.1371/journal.pone.0231710)
Supplement: S1 Data — (PDF) [file pone.0231710.s001.pdf]

Dato:
ID nummer:

Alder:

Kjønn:
□ Kvinne
□ Mann

1. Hva er din høyeste fullførte utdanning?
□ Grunnskole/folkeskole
□ Grunnkurs/1-2 årig utdannelse etter grunnskole
□ Videregående/gymnas/yrkesskole (3-årig)
□ Fagbrev
□ Universitet/høyskole på 4 år eller mindre
□ Universitet/høyskole på mer enn 4 år
□ Annet: ____

2. Har du vært i arbeid de siste 12 måneder?
□ Nei
□ Ja

3. Vi antar at din arbeidsevne, da den var best, vurderes med 10 poeng. Hvilket poengtall ville du gitt din nåværende arbeidsevne?

0 1 2 3 4 5 6 7 8 9 10
□ □ □ □ □ □ □ □ □ □ □

4. Har du vært sykemeldt i løpet av de siste 12 månedene?
□ Nei
□ Ja

5. Har en lege noen gang gitt deg diagnosen astma?
□ Nei
□ Ja

Andre sykdommer eller plager:
6. Har en lege noen gang fortalt deg at du har kronisk obstruktiv lungesykdom (KOLS)?
□ Nei
□ Ja

7. Annen kronisk lungesykdom enn astma eller KOLS?
□ Nei
□ Ja

8. Hvor ofte spiser du:

	0-3 ganger pr mnd	1-3 ganger pr uke	4-6 ganger pr uke	1 gang pr dag	2 ganger eller mer pr dag
Frukt/bær	□	□	□	□	□
Grønnsaker	□	□	□	□	□
Sjokolade/godteri	□	□	□	□	□
Pølser/hamburgere	□	□	□	□	□
Fet fisk (laks, ørret, sild, makrell, uer som pålegg/middag)	□	□	□	□	□

9. Hvor ofte mosjonerer/trener du? (Ta et gjennomsnitt)
□ Aldri
□ 2-3 ganger pr uke
□ Mindre enn 1 gang pr uke
□ 1 gang pr uke
□ Omtrent daglig (4-7 ganger pr uke)

10. Hvis du trener 1 gang pr uke eller mer:
Hvor hardt mosjonerer/trener du?
□ Tar det rolig uten å bli andpusten eller svett
□ Tar det så hardt at jeg blir andpusten og/eller svett
□ Tar meg nesten helt ut

11. Hvor lenge pleier du å trene? (Ta et gjennomsnitt)
□ Mindre enn 15 minutter
□ 15-29 minutter
□ 30 minutter til 1 time
□ Mer enn 1 time

12. vekt: kg
13. høyde: cm

14. Røyker du daglig (gjelder selv om du kun røyker noen få sigaretter, sigarer eller pipe daglig)?
□ Nei
□ Ja

15. Røyker du bare av og til (ikke daglig, men helger, festrøyking eller liknende)?
□ Nei
□ Ja

16. Har du røykt tidligere?
□ Nei
□ Ja
