# Supplementary material for: Lifestyle, sick leave and work ability among Norwegian employees with asthma—A population-based cross-sectional survey conducted in Telemark County, Norway
Source: PLoS One. 2020 Apr 17;15(4):e0231710. doi: 10.1371/journal.pone.0231710 (PMC7164599; doi:10.1371/journal.pone.0231710)
Supplement: S2 Data — (DOCX) [file pone.0231710.s002.docx]

Date:

ID number:

Age:

Gender:

□ Female

□ Male

1. What is your highest level education?

□ Elementary school/grade school

□ Basic courses/1-2 year(s) of education after elementary school

□ Secondary/high school/vocational school (3-years)

□ Certificate

□ University/College - 4 years or less

□ University/College - more than 4 years

□ Other: _____________________________________________

2. Have you been engaged in paid work for the past 12 months?

□ No

□ Yes

3. We assume that your work ability, when it was at its best would rate 10 points. How many points would you give to rate your work ability?

0 1 2 3 4 5 6 7 8 9 10

□ □ □ □ □ □ □ □ □ □ □

4. Have you been on sick leave over the course of the last 12 months?

□ No

□ Yes

5. Has a physician ever diagnosed you with asthma?

□ No

□ Yes

Other disease or illnesses:

6. Has a doctor ever told you that you have chronic obstructive pulmonary disease (COPD)?

□ No

□ Yes

7. Has a physician ever diagnosed you with any chronic lung disease other than chronic obstructive lung disease or asthma?

□ No

□ Yes

8. How often do you usually eat these foods? Make a cross in the box

|  | 0-3 times per month | 1-3 times per week | 4-6 times per week | 1 time  per day | 2 times or more  per day |
| --- | --- | --- | --- | --- | --- |
| Fruit/berries | □ | □ | □ | □ | □ |
| Vegetables | □ | □ | □ | □ | □ |
| Chocolate/candies | □ | □ | □ | □ | □ |
| Sausages/hamburgers | □ | □ | □ | □ | □ |
| Fatty fish  (salmon, trout, herring, mackerel, redfish as toppings at dinner) | □ | □ | □ | □ | □ |

9. How often do you exercise? (Give an average)

□ Never

□ 2-3 times per week

□ Less than 1 time per week

□ 1 time per week

□ Daily/almost daily (4-7) times per week

10. If you exercise once per week or more:

How hard do you exercise?

□ Take it easy without getting sweaty or out of breath

□ I am out of breath and/or sweaty

□ I am almost exhausted

11. For how long do you usually work out? (Give an average)

□ Less than 15 minutes

□ 15-29 minutes

□ 30 minutes to 1 hour

□ More than 1 hour

12. weight: kg

13. height: cm

14. Do you smoke every day (even if you only smoke a few cigarettes, cigars or a pipe daily)?

□ No

□ Yes

15. Do you smoke occasionally (not daily, but weekends, party smoking or the like)?

□ No

□ Yes

16. If not, have you smoked in the past?

□ No

□ Yes
