# Supplementary material for: Lifestyle, sick leave and work ability among Norwegian employees with asthma—A population-based cross-sectional survey conducted in Telemark County, Norway
Source: PLoS One. 2020 Apr 17;15(4):e0231710. doi: 10.1371/journal.pone.0231710 (PMC7164599; doi:10.1371/journal.pone.0231710)
Supplement: Attachment — Submitted filename: PONE-D-19-34978.docx [file pone.0231710.s004.docx]

**PONE-D-19-34978**

Thank you for the opportunity to review this paper. The manuscript is technically sound, the data support the conclusions and presented in an intelligible fashion and written in standard English. The paper gives a novelty insight how multiple lifestyle risk factors are associated to asthma and sick-leave. I would suggest the following – in order to make these interactions more understandable to your audience, I would suggest you make a diagram to illustrate these interactions. Also, I would suggest to always use the same wording throughout the whole manuscript- multiple lifestyle risk factors.
