# Supplementary material for: Lifestyle, sick leave and work ability among Norwegian employees with asthma—A population-based cross-sectional survey conducted in Telemark County, Norway
Source: PLoS One. 2020 Apr 17;15(4):e0231710. doi: 10.1371/journal.pone.0231710 (PMC7164599; doi:10.1371/journal.pone.0231710)
Supplement: Attachment — Submitted filename: 2020-03.25.Rebuttal letter PlosOne.MaritMDB.docx [file pone.0231710.s005.docx]

Dear PlosOne Editors,

Thank you for the opportunity to revise our manuscript. We are pleased that the reviewer found our article interesting and novel in its field. We would like to thank the reviewer for their useful comments, which we believe have improved our manuscript substantially. We address her/his comments below and have made revisions to the manuscript (we refer to pages and paragraphs in the “track changes” version). Our revised manuscript totals 3697 words (including the abstract), which we hope is acceptable.

Additional requirements:

1. Please ensure that your manuscript meets PLOS ONE's style requirements, including those for file naming. The PLOS ONE style templates can be found at

<http://www.journals.plos.org/plosone/s/file?id=wjVg/PLOSOne_formatting_sample_main_body.pdf> and <http://www.journals.plos.org/plosone/s/file?id=ba62/PLOSOne_formatting_sample_title_authors_affiliations.pdf>

*Answer: we have formatted according to PLOS ONE’s requirements.*

2. Please provide additional details regarding participant consent. In the ethics statement in the Methods and online submission information, please ensure that you have specified (1) whether consent was informed and (2) what type you obtained (for instance, written or verbal). If your study included minors, state whether you obtained consent from parents or guardians. If the need for consent was waived by the ethics committee, please include this information.

*Answer: the participant consent in this study was informed and written. We have included this information. See page 9.*

3. Please include additional information regarding the survey or questionnaire used in the study and ensure that you have provided sufficient details that others could replicate the analyses. For instance, if you developed a questionnaire as part of this study and it is not under a copyright more restrictive than CC-BY, please include a copy, in both the original language and English, as Supporting Information.

*Answer: we have included both Norwegian and English version of the questionnaire. Only questions used in this study have been included. See S1 and S2.*

4. We note that you have included the phrase “data not shown” in your manuscript. Unfortunately, this does not meet our data sharing requirements. PLOS does not permit references to inaccessible data. We require that authors provide all relevant data within the paper, Supporting Information files, or in an acceptable, public repository. Please add a citation to support this phrase or upload the data that corresponds with these findings to a stable repository (such as Figshare or Dryad) and provide and URLs, DOIs, or accession numbers that may be used to access these data. Or, if the data are not a core part of the research being presented in your study, we ask that you remove the phrase that refers to these data.

*Answer: we have removed the sentence containing “data not shown”. We consider this was not an essential part of the manuscript and decided to exclude this sentence. See page 16.*

5. We note that you have indicated that data from this study are available upon request. PLOS only allows data to be available upon request if there are legal or ethical restrictions on sharing data publicly. For information on unacceptable data access restrictions, please see <http://journals.plos.org/plosone/s/data-availability#loc-unacceptable-data-access-restrictions>.

In your revised cover letter, please address the following prompts:

a) If there are ethical or legal restrictions on sharing a de-identified data set, please explain them in detail (e.g., data contain potentially identifying or sensitive patient information) and who has imposed them (e.g., an ethics committee). Please also provide contact information for a data access committee, ethics committee, or other institutional body to which data requests may be sent.

*Answer: We consider our underlying minimal data set to contain sensitive data, and also potentially identifiable individuals. Sharing restrictions on the minimal data set are imposed from: The Regional Committee for Medical and Health Professional Research Ethics in South-east Norway (Study ID: REC 2012/1665), The Norwegian Data Inspectorate and the Telemark Hospital Department of Research and Development. However, data may be shared for researchers who meet the criteria for access to confidential data upon request to the head of the Telemark-Study steering committee: Dr. Trude K. Fossum, Department of Occupational and Environmental Medicine, Telemark Hospital, Post box 2900 Kjøbekk, 3710 Skien. E-mail:* [*fotr@sthf.no*](mailto:fotr@sthf.no)

*The minimal data set identification: Minimal.Lifestyle.WAS.SL.asthma.sav*

b) If there are no restrictions, please upload the minimal anonymized data set necessary to replicate your study findings as either Supporting Information files or to a stable, public repository and provide us with the relevant URLs, DOIs, or accession numbers. Please see <http://www.bmj.com/content/340/bmj.c181.long> for guidelines on how to de-identify and prepare clinical data for publication. For a list of acceptable repositories, please see <http://journals.plos.org/plosone/s/data-availability#loc-recommended-repositories>.

We will update your Data Availability statement on your behalf to reflect the information you provide.

[Note: HTML markup is below. Please do not edit.]

Reviewers' comments:

Reviewer's Responses to Questions

**Comments to the Author**

1. Is the manuscript technically sound, and do the data support the conclusions?

The manuscript must describe a technically sound piece of scientific research with data that supports the conclusions. Experiments must have been conducted rigorously, with appropriate controls, replication, and sample sizes. The conclusions must be drawn appropriately based on the data presented.

Reviewer #1: Yes

2. Has the statistical analysis been performed appropriately and rigorously?

Reviewer #1: Yes

3. Have the authors made all data underlying the findings in their manuscript fully available?

The [PLOS Data policy](http://www.plosone.org/static/policies.action#sharing) requires authors to make all data underlying the findings described in their manuscript fully available without restriction, with rare exception (please refer to the Data Availability Statement in the manuscript PDF file). The data should be provided as part of the manuscript or its supporting information, or deposited to a public repository. For example, in addition to summary statistics, the data points behind means, medians and variance measures should be available. If there are restrictions on publicly sharing data—e.g. participant privacy or use of data from a third party—those must be specified.

Reviewer #1: Yes

4. Is the manuscript presented in an intelligible fashion and written in standard English?

PLOS ONE does not copyedit accepted manuscripts, so the language in submitted articles must be clear, correct, and unambiguous. Any typographical or grammatical errors should be corrected at revision, so please note any specific errors here.

Reviewer #1: Yes

5. Review Comments to the Author

Please use the space provided to explain your answers to the questions above. You may also include additional comments for the author, including concerns about dual publication, research ethics, or publication ethics. (Please upload your review as an attachment if it exceeds 20,000 characters)

Reviewer #1: Thank you for the opportunity to review this paper. The manuscript is technically sound, the data support the conclusions and presented in an intelligible fashion and written in standard English. The paper gives a novelty insight how multiple lifestyle risk factors are associated to asthma and sick-leave. I would suggest the following – in order to make these interactions more understandable to your audience, I would suggest you make a diagram to illustrate these interactions. Also, I would suggest to always use the same wording throughout the whole manuscript- multiple lifestyle risk factors.

*Answer: Thank you for these important comments. We have added a figure (Fig 2) in the manuscript illustrating the hypothesized interaction between lifestyle risk factors- asthma- sick leave/work ability. See page 8 and attached figure. We have obtained license from Springer Nature since the figure is inspired by an article from one of their Journals; Int Arch Occup Environ Health. 2019;92(6):855-64. License Number 4790660096986, License date March 16, 2020.*

*We have also corrected the wording to make the whole manuscript consistent with the wording of “multiple lifestyle risk factors”.*

One behalf of the authors,

Marit Müller De Bortoli
